# Anti-IL-2 Treatment Impairs the Expansion of T_reg_ Cell Population during Acute Malaria and Enhances the Th1 Cell Response at the Chronic Disease

**DOI:** 10.1371/journal.pone.0029894

**Published:** 2012-01-17

**Authors:** Cláudia A. Zago, Karina R. Bortoluci, Luiz R. Sardinha, Fernando D. Pretel, Sheyla I. Castillo-Méndez, Ana Paula Freitas do Rosário, Meire I. Hiyane, Sandra M. Muxel, Sérgio M. Rodriguez-Málaga, Ises A. Abrahamsohn, José M. Álvarez, Maria Regina D'Império Lima

**Affiliations:** 1 Departamento de Imunologia, Instituto de Ciências Biomédicas, Universidade de São Paulo, São Paulo, São Paulo, Brasil; 2 Departamento de Ciências Biológicas (Campus Diadema) and Centro de Terapia Celular e Molecular (CTC-Mol), Universidade Federal de São Paulo, São Paulo, São Paulo, Brasil; 3 Instituto Israelita de Ensino e Pesquisa Albert Einstein, São Paulo, São Paulo, Brasil; University of Palermo, Italy

## Abstract

*Plasmodium chabaudi* infection induces a rapid and intense splenic CD4^+^ T cell response that contributes to both disease pathogenesis and the control of acute parasitemia. The subsequent development of clinical immunity to disease occurs concomitantly with the persistence of low levels of chronic parasitemia. The suppressive activity of regulatory T (T_reg_) cells has been implicated in both development of clinical immunity and parasite persistence. To evaluate whether IL-2 is required to induce and to sustain the suppressive activity of T_reg_ cells in malaria, we examined in detail the effects of anti-IL-2 treatment with JES6-1 monoclonal antibody (mAb) on the splenic CD4^+^ T cell response during acute and chronic *P. chabaudi* AS infection in C57BL/6 mice. JES6-1 treatment on days 0, 2 and 4 of infection partially inhibits the expansion of the CD4^+^CD25^+^Foxp3^+^ cell population during acute malaria. Despite the concomitant secretion of IL-2 and expression of high affinity IL-2 receptor by large CD4^+^ T cells, JES6-1 treatment does not impair effector CD4^+^ T cell activation and IFN-γ production. However, at the chronic phase of the disease, an enhancement of cellular and humoral responses occurs in JES6-1-treated mice, with increased production of TNF-α and parasite-specific IgG2a antibodies. Furthermore, JES6-1 mAb completely blocked the *in vitro* proliferation of CD4^+^ T cells from non-treated chronic mice, while it further increased the response of CD4^+^ T cells from JES6-1-treated chronic mice. We conclude that JES6-1 treatment impairs the expansion of T_reg_ cell population during early *P. chabaudi* malaria and enhances the Th1 cell response in the late phase of the disease.

## Introduction

The asexual blood stages of the *Plasmodium ssp.* are responsible for the pathology and morbidity caused by malaria, an infectious disease that remains a major devastating illness afflicting 350 to 500 million people annually and resulting in more than 1 million deaths per year [1]. Among the cell populations involved in the immune response to the blood stages of malaria, effector Th1 cells are thought to play a key role in both disease protection and pathogenesis [2], [3], [4]. Thus, an appropriate regulatory balance between protective immune responses and immune mediated pathology is required for a favorable outcome of infection [5]. The suppressive activity of regulatory T (T_reg_) cells has been implicated in the development of clinical immunity to disease known as premunition, which occurs concomitantly with persistence of low parasite burdens rather than sterilizing immunity [5]. However, despite their relevance, the molecular pathways required to induce and to sustain the suppressive activity of T_reg_ cells in malaria are still poorly characterized.

In the blood stage malaria caused by the rodent parasite, *Plasmodium chabaudi*, the spleen CD4^+^ T cell population expands quickly, secretes considerable amounts of interferon-γ (IFN-γ) and helps B cells to produce high quantities of polyclonal immunoglobulin (Ig) M and IgG2a [6], [7], [8]. Both IFN-γ [9] and acute phase antibodies [10] have been implicated in the initial control of the parasite. Effector Th1 cells also contribute to pathogenesis in acute *P. chabaudi* malaria because mice lacking IFN-γ or deprived of this cell population have attenuated symptoms [11]. As the disease progresses, the majority of lymphocytes activated during early infection are eliminated by apoptosis [12], giving the opportunity to the development of a large pool of effector-memory CD4^+^ T cells that cooperate with B cells in the production of parasite-specific high-affinity antibodies and have the capacity to secrete IFN-γ upon stimulation [13]. Similar to humans infected with *Plasmodium falciparum*, the development clinical immunity to *P. chabaudi* malaria occurs simultaneously with persistence of low levels of chronic parasitemia [14], and T_reg_ cells have also been implicated in both processes [5]. The cooperation between high-affinity parasite-specific IgG and memory Th1 cells is required for complete parasite clearance after 2–3 months of infection and also for acquisition of full protective immunity against reinfection [14], [15].

In contrast to the many studies addressing the role of CD4^+^ T cells in protection against *P. chabaudi* malaria, little is known about the molecular mechanisms responsible for CD4^+^ T cell proliferation, differentiation and regulation. IL-2 has multiple and opposing activities contributing to both the induction and the control of immune responses [16], [17]. Both activated and regulatory CD4^+^ T cells express CD25, the α chain of the high-affinity IL-2 receptor (IL-2R) that combines with the IL-2R β chain (CD122) and the common γ chain (γc or CD132). While activated CD4^+^ T cells can produce their own IL-2, T_reg_ cells depend on paracrine IL-2 for their generation and maintenance and for the exertion of their suppressive functions [18]. Thus, although IL-2 was first identified as a potent T cell growth factor [19] that also displays pro-apoptotic activity [20], the main non-redundant activity of IL-2 is to promote T cell tolerance and homeostasis [21], [22]. Moreover, IL-2 is required for effector Th1 and Th2 cell differentiation, provides a competitive advantage to T cells, resulting in optimal survival and performance of memory cells, and inhibits the development of inflammatory Th17 cells [16].

In the present study, we analyzed in detail the effects of anti-IL-2 treatment with JES6-1 monoclonal antibody (JES6-1 mAb) on the CD4^+^ T cell response to *P. chabaudi*. This mAb has been shown to block the CD8^+^ T cell response to IL-2 *in vitro* via the low-affinity IL-2R βγ, apparently for biding to an IL-2 site that is crucial for interaction with CD122 but less crucial for binding to CD25 (high-affinity IL-2Rαβγ) [23]. Because IL-2 bound to JES6-1 mAb has extended *in vivo* half-time and retains the ability to interact with the high-affinity IL-2R, injection of a premixed 2∶1 molar ratio of IL-2/JES6-1 mAb complexes has been used to potentiate IL-2 signaling and induce expansion of the T_reg_ cell population [24]. Thus, analyzing the effects of JES6-1 treatment on *P. chabaudi* malaria contributes to the efforts to understand the molecular mechanisms responsible for activation and regulation of the CD4^+^ T cell response to *Plasmodium* aiming to ameliorate the outcome of the disease.

## Results

### Expression of IL-2R α (CD25) and β (CD122) chains in the splenic CD4^+^ T cell response to *P. chabaudi* malaria

**Figure 1:**
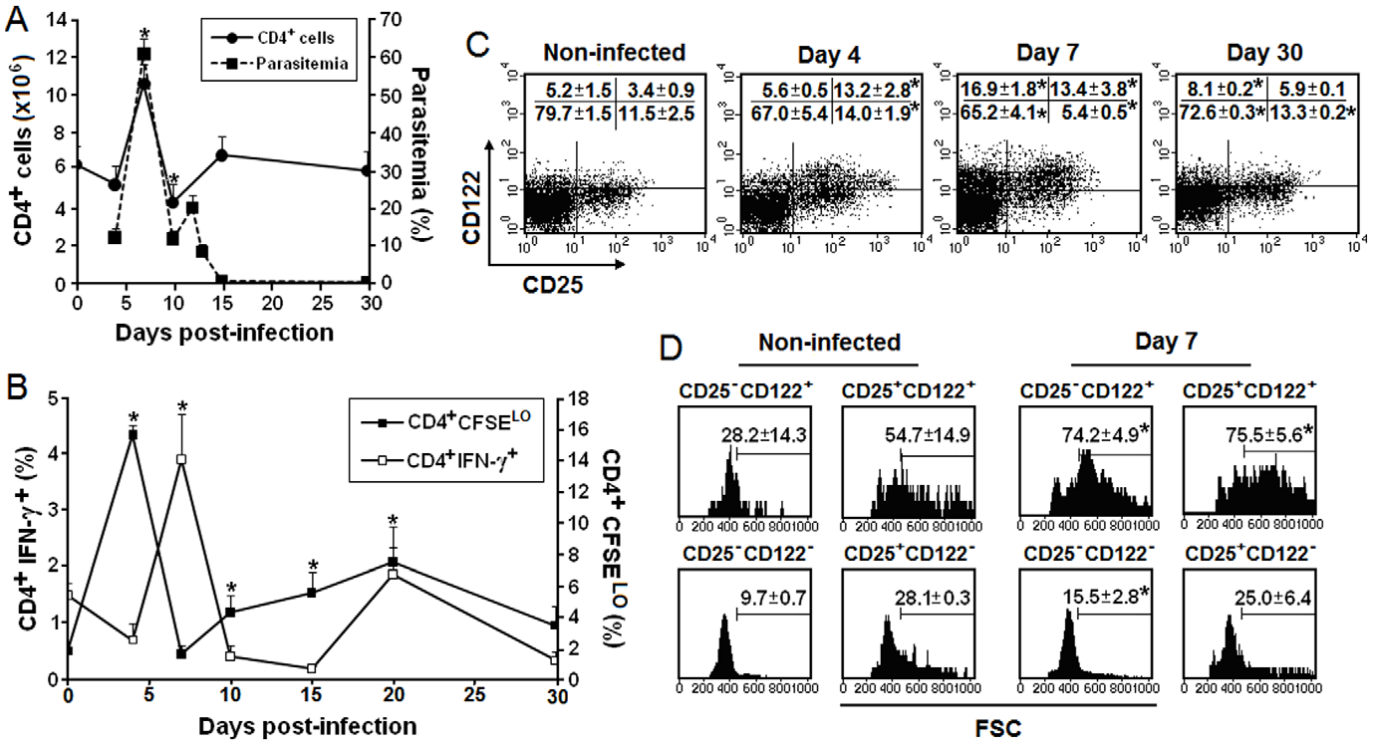
Expression of IL-2R α and ß chains in splenic CD4^+^ T cells during *P. chabaudi* malaria. (A) Parasitemia curves and CD4^+^ T cell numbers per spleen were evaluated in C57BL/6 mice infected with 10^6^ PRBC (mean ± SD, n = 4). (B) Basal (non-stimulated) proliferation and IFN-γ production in infected mice. Percentages of replicating (CSFE^LO^) CD4^+^ T cells and CD4^+^IFN-γ^+^ cells are shown (mean ± SD, n = 4). (C) On days 4, 7 and 30 p.i., CD25 and CD122 expression was analyzed in gated CD4^+^ T cells. Numbers inside dot plots refer to means ± SD (n = 3–4) of cell percentages in each gate. (D) On day 7 p.i., CD25^−^CD122^+^, CD25^+^CD122^+^, CD25^−^CD122^−^ and CD25^+^CD122^−^ cells in gated CD4^+^ T cells of the same groups of mice were analyzed according to cell size (FSC). Numbers inside histograms refer to means ± SD (n = 3–4) of large cell percentages. In A–D, *p<0.05, compared to non-infected mice (day 0). Dot plots and histograms show a representative mouse of each group. Data are representative of three separate experiments.

The infection of C57BL/6 mice with 10^6^ parasitized red blood cells (PRBC) resulted in a rapid increase in splenic CD4^+^ T cell numbers, attaining maximum levels concomitant with the peak of parasitemia on day 7 post-infection (p.i.) (**Fig. 1A**). The increase of the CD4^+^ T cell population was accompanied by intense proliferation and culminated in a prominent but short-lasting peak of IFN-γ production (**Fig. 1B**). As recently shown, the great majority of proliferating and IFN-γ-producing CD4^+^ cells in the spleen during the early infection are class II MHC-restricted CD4^+^ T cells and not NKT cells [13]. On day 10 p.i., in parallel with the control of parasitemia, CD4^+^ T cell numbers per spleen abruptly decreased to values lower than those of non-infected mice (**Fig. 1A**). The normalization of this population occurred concomitantly with a second wave of CD4^+^ T cell proliferation that peaked on day 20 p.i. and decreased thereafter (**Fig. 1B**).

Along with the proliferative response to infection, there was a notable increase in CD122 expression, which gave evidence of two different CD4^+^ T cell subsets (**Fig. 1C**). The CD25^+^CD122^+^ subset reached its maximum on days 4 and 7 p.i. before decreasing. The CD25^−^CD122^+^ subset appeared later, being first observed on day 7 p.i. and still detectable on days 15 p.i. (data not shown) and 30 p.i. when it corresponded to 13.6% and 8.1% of CD4^+^ T cells, respectively. On day 7 p.i., both CD4^+^CD122^+^ cell populations had a large size, whereas the majority of CD4^+^CD25^+^CD122^−^ cells were smaller (**Fig. 1D**). CD4^+^CD25^+^CD122^+^ cells from 4-day infected mice and CD4^+^CD25^−^CD122^+^ cells from 15-day infected mice were also large, whereas CD4^+^CD25^+^CD122^−^ cells from 30-day infected mice were smaller (data not shown). As expected, CD4^+^CD25^+^CD122^LO/-^ cells, a phenotype characteristic of T_reg_ cells, were also observed in non-infected mice. These results show that splenic CD4^+^ T cells that respond to acute *P. chabaudi* malaria express the IL-2R β chain, but only a subset co-express the α chain. During chronic infection, the majority of activated CD4^+^ T cells present only the IL-2R β chain.

### Secretion of IL-2 and expression of activation markers by splenic CD4^+^ T cells during *P. chabaudi* malaria

**Figure 2:**
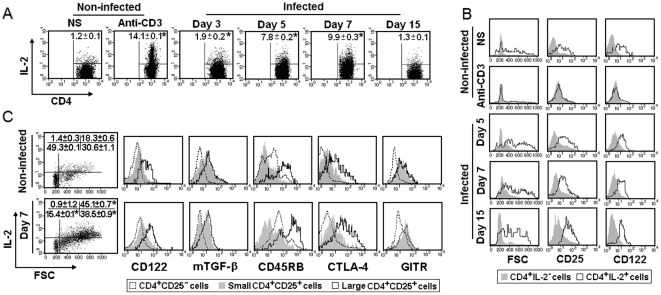
Secretion of IL-2 and expression of activation markers by splenic CD4^+^ T cells during *P. chabaudi* malaria. (A) IL-2 secretion was analyzed in gated CD4^+^ T cells obtained from C57BL/6 mice on days 3, 5, 7 and 15 p.i. with 10^6^ PRBC. Numbers inside dot plots refer to means ± SD (n = 3–4) of cell percentages in the upper right gate. (B) Cell size (FSC) and expression of CD25 and CD122 were evaluated in gated CD4^+^IL-2^−^ and CD4^+^IL-2^+^ cells of the same groups of mice. (C) On day 7 p.i., CD122, mTGF-β, CD45RB, CTLA-4 and GITR expression was analyzed in gated CD4^+^CD25^+^ cells, subdivided into small and large cells. Numbers inside dot plots refer to means ± SD (n = 3–4) of cell percentages in each gate. The mean fluorescence intensity (MFI) of CD4^+^CD25^−^ cells (controls) stained with mAb to mTGF-ß and GITR was comparable to respective isotopic controls (data not shown). In A–C, there was a significant difference (*p<0.05) between experimental conditions and non-stimulated (NS) cells from non-infected mice. Cells from non-infected mice stimulated with anti-CD3 mAb were used as positive controls. Dot plots and histograms show a representative mouse of each group. Data are representative of three separate experiments.

Concomitantly with the high expression of IL-2R, the percentages of IL-2-secreting CD4^+^ T cells progressively increased from day 3 to 7 p.i., decreasing to levels similar to those of non-infected mice on day 15 p.i. (**Fig. 2A**). IL-2-secreting CD4^+^ T cells were large in size and expressed high amounts of CD25 and CD122 (**Fig. 2B**). The majority of large CD4^+^CD25^+^ cells in 7-day infected mice produced IL-2 (**Fig. 2C**). Small CD4^+^CD25^+^ cells had a phenotype typical of T_reg_ cells in both groups of mice. When compared to CD4^+^CD25^−^ cells, these cells expressed significantly higher levels of mTGF-β, CTLA-4 and GITR, slightly higher levels of CD122 and lower levels of CD45RB. In contrast, large CD4^+^CD25^+^ cells had a phenotype characteristic of activated cells, differing from small CD4^+^CD25^+^ cells by the extremely high expression of CD122, CD45RB and CTLA-4. These results show that IL-2 is produced during acute *P. chabaudi* malaria by large CD4^+^ T cells that express high levels of IL-2R, together with several other activation markers.

### Effects of JES6-1 treatment on the early CD4^+^ T cell response to *P. chabaudi* malaria

**Figure 3:**
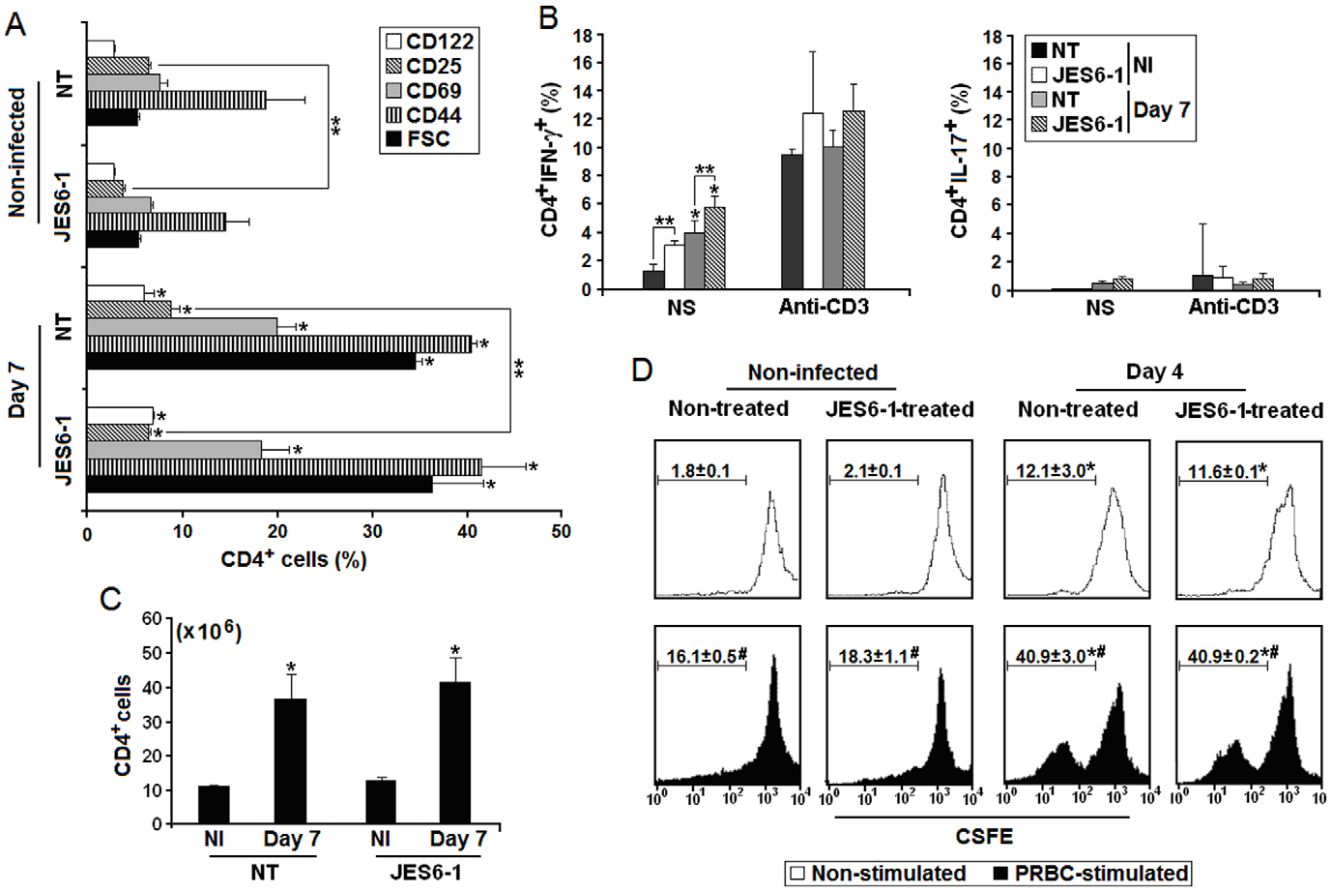
Effects of JES6-1 treatment on the early CD4^+^ T cell response to *P. chabaudi* malaria. (A) C57BL/6 mice were treated with JES6-1 mAb on days 0, 2 and 4 p.i. with 10^6^ PRBC. On day 7 p.i., splenic CD4^+^ T cells were analyzed for CD122, CD25, CD69 and CD44 expression and cell size (FSC). Data show gated CD4^+^ T cells expressing high levels of activation markers and large size (n = 3–4). (B) Non-stimulated (basal) and anti-CD3 mAb stimulated IFN-γ and IL-17 production was evaluated in CD4^+^ T cells from the same groups of mice. (C) Numbers of CD4^+^ T cells per spleen were determined in the same groups of mice. (D) On day 4 p.i., PRBC-stimulated CD4^+^ T cell proliferation was analyzed *in vitro* in the presence or absence of JES6-1 mAb. Histograms show CFSE fluorescence in gated CD4^+^ T cells. The means ± SD (n = 3–4) of the percentages of replicating (CFSE^LO^) cells are shown. In A–D, significant differences compared experimental conditions *p<0.05 with cells from non-infected (NI) mice; **p<0.05 with cells from non-treated (NT) mice; and #p<0.05 with non-stimulated cells. Data are representative of three separate experiments.

C57BL/6 mice treated with JES6-1 monoclonal antibodies (mAb) on days 0, 2 and 4 p.i. with 10^6^ PRBC were analyzed according to expression of IL-2Rα and β chains and the activation markers CD69 (early activation-induced C-type lectin) and CD44 (cell-cell contact adhesion molecule). The production of IL-17 was also evaluated because anti-IL-2 treatment could shift the immune response towards the Th17 profile, due to the negative control exerted by IL-2 over development of inflammatory Th17 cells [16]. Based on the percentages of CD4^+^ cells showing high expression of CD122, CD25, CD69 and CD44 and large size, JES6-1 mAb had negligible effects on CD4^+^ T cell activation with only a minor inhibition of CD25 expression (**Fig. 3A**). The cytokine pattern of CD4^+^ T cells responding to acute infection remained unchanged where the production of IFN-γ predominated over IL-17 secretion (**Fig. 3B**). Nevertheless, JES6-1 treatment resulted in a significant increase in basal (non-stimulated) IFN-γ production by CD4^+^ T cells in both non-infected mice and 7-day infected mice. The CD4^+^ T cell numbers per spleen (**Fig. 3C**) and the capacity to control parasitemia (data not shown) were not affected by JES6-1 treatment. Therefore, we also evaluated the *in vitro* effects of the JES6-1 mAb on PRBC-stimulated CD4^+^ T cell proliferation. Cells obtained on day 4 p.i. were analyzed because this is the peak of the *in vitro* proliferative response to PRBC during acute infection [13]. As observed *in vivo*, the JES6-1 mAb had no inhibitory effect on *in vitro* CD4^+^ T cell proliferation (**Fig. 3D**). These results show that JES6-1 treatment does not significantly alter the CD4^+^ T cell activation, proliferation and IFN-γ production during acute *P. chabaudi* malaria.

### Effects of JES6-1 treatment on the CD4^+^CD25^+^FoxP3^+^ cell population during *P. chabaudi* malaria

**Figure 4:**
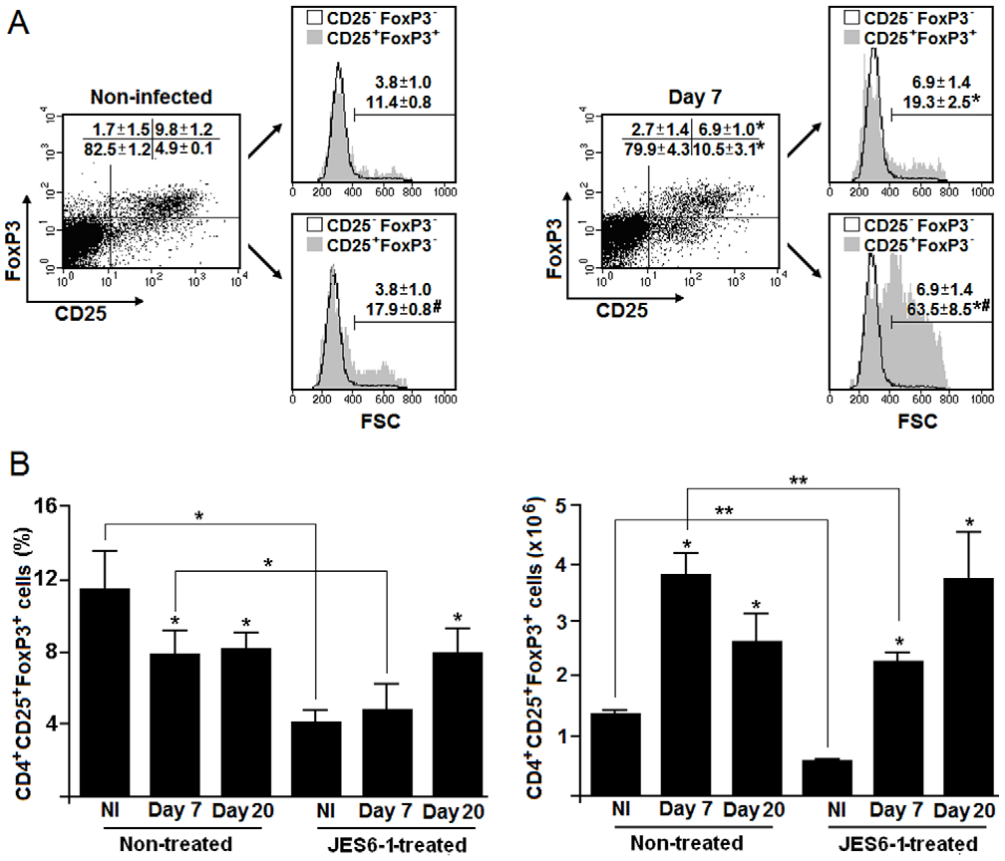
Effects of JES6-1 treatment on the splenic CD4^+^CD25^+^FoxP3^+^ cell population during *P. chabaudi* malaria. (A) C57BL/6 mice were infected with 10^6^ PRBC. On day 7 p.i., CD4^+^ T cells were analyzed for CD25 and FoxP3 expression and cell size (FSC). Dot plots represent gated CD4^+^ T cells. Dot plots and histograms show a representative mouse from each group. Numbers inside dot plots refer to means ± SD (n = 4) of cell percentages in each gate. Histograms show gated CD4^+^CD25^+^FoxP3^+^ and CD4^+^CD25^+^FoxP3^−^ cells in relation to CD4^+^CD25^−^FoxP3^−^ cells. Numbers inside histograms refer to means ± SD (n = 4) of large cell percentages. (B) On days 0, 2 and 4 p.i., C57BL/6 mice were treated with JES6-1 mAb. Data represent the means ± SD (n = 4) of CD4^+^CD25^+^FoxP3^+^ cell percentages and numbers per spleen on days 7 and 20 of infection. In A–B, significant differences compared experimental conditions *p<0.05 with cells from non-infected (NI) mice; **p<0.05 with cells from non-treated (NT) mice; and #p<0.05 with CD25^+^FoxP3^+^ cells. Data are representative of two separate experiments.

The expression of FoxP3 was evaluated in splenic CD4^+^CD25^+^ cells from C57BL/6 mice on days 7 and 20 p.i. with 10^6^ PRBC. In non-infected mice, the great majority of CD4^+^CD25^+^ cells expressed FoxP3, a phenotype characteristic of T_reg_ cells (**Fig. 4A**). On day 7 p.i., although the majority of large cells were comprised in the CD4^+^CD25^+^FoxP3^−^ subpopulation, there was an increase of 69.3% in the large CD4^+^CD25^+^FoxP3^+^ subpopulation. When considering cell number per spleen, there was a 3-fold increase in the CD4^+^CD25^+^FoxP3^+^ cell population on day 7 p.i. followed by a reduction of 40.7% by day 20 p.i. (**Fig. 4B**). JES6-1 treatment partially reduced CD4^+^CD25^+^FoxP3^+^ cell numbers per spleen in non-infected and 7-day infected mice. The involvement of IL-2 in the maintenance/expansion of the T_reg_ cell population was also evidenced by data showing a significant decrease in CD4^+^CD25^+^FoxP3^+^ cell percentages in non-infected and 7-day infected mice treated with JES6-1 mAb compared to non-treated controls.

### Effects of JES6-1 treatment on CD4^+^ T cell phenotype and proliferative response during chronic *P. chabaudi* malaria

**Figure 5:**
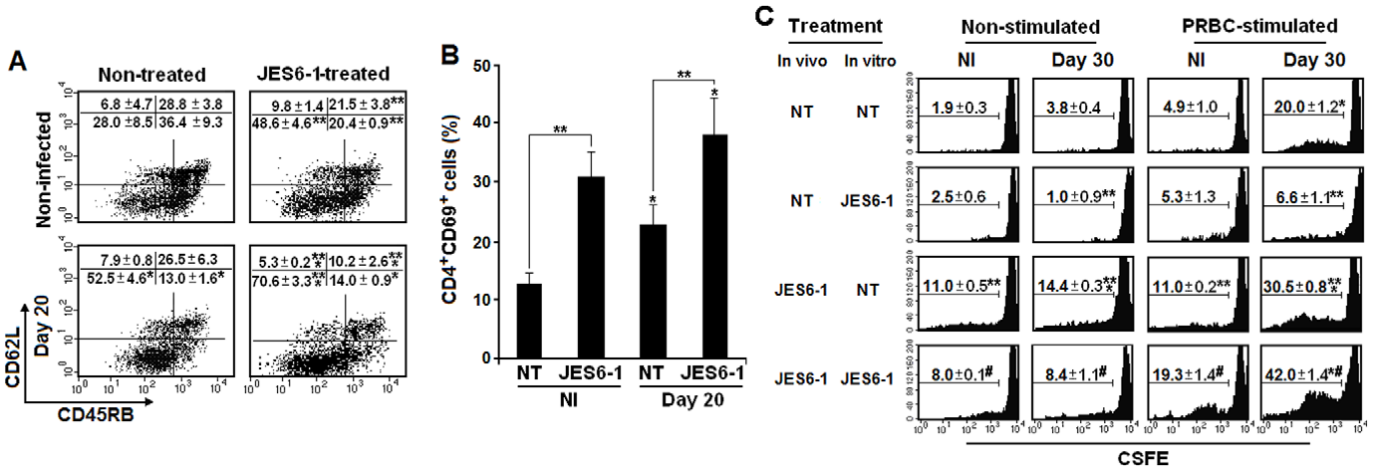
Effects of JES6-1 treatment on splenic CD4^+^ T cell phenotype and proliferative response to PRBC during chronic *P. chabaudi* malaria. (A) C57BL/6 mice were treated with JES6-1 mAb on days 0, 2 and 4 p.i. with 10^6^ PRBC. On day 20 p.i., CD62L and CD45RB expression was evaluated in gated CD4^+^ T cells. Numbers inside dot plots refer to means ± SD (n = 3–4) of cell percentages in each gate. (B) CD69 expression was analyzed in gated CD4^+^ T cells from the same groups of mice. (C) *In vitro* proliferative response of PRBC-stimulated CD4^+^ cells cultured in the presence or absence of JES6-1 mAb. CD4^+^ T cells were obtained from 30-day infected mice treated *in vivo* or not with JES6-1 mAb on days 0, 2 and 4 of infection. Histograms show CFSE fluorescence in gated CD4^+^ T cells. The means ± SD (n = 3–4) of the percentages of replicating (CFSE^LO^) cells are shown. In A–C, significant differences compared experimental conditions *p<0.05 with cells from non-infected (NI) mice; **p<0.05 with non-treated (NT) mice or cells; and # p<0.05 with non-treated (NT) cells from JES6-1-treated mice. Data are representative of two separate experiments.

Because IL-2 secreted during acute *P. chabaudi* malaria might influence the late phase of the immune response either directly or through its effects on T_reg_ cells, we evaluated the effects of JES6-1 treatment on the CD4^+^ T cell phenotype and proliferative response on days 20 and 30 p.i., respectively. These time points coincided with the peak and the end of the second wave of CD4^+^ T cell proliferation, respectively (**Fig. 1B**). Twenty days after the beginning of JES6-1 treatment, there was no significant difference in CD4^+^ T cell numbers per spleen between non-infected mice and infected mice (data not shown). However, JES6-1 treatment resulted in additive increases in percentages of CD4^+^CD62L^LO^CD45RB^LO^ (effector-memory) cells and CD4^+^CD69^+^ (activated) cells (**Fig. 5A and 5B**). Mice treated with anti-βgalactosidase isotype control (GL117 mAb) did not differ from non-treated mice (data not shown). Non-stimulated (basal) and PRBC-stimulated CD4^+^ T cell proliferation was also increased in JES6-1-treated mice, a phenomenon observed in non-infected and infected mice (**Fig. 5C**). The CD8^+^ T cell proliferation in response to PRBC was also enhanced in both groups of JES6-1-treated mice (3.7±0.1% and 13.0±1.6% of CSFE^LO^ cells, p<0.05) compared to non-treated mice (1.9±0.1% and 4.8±0.4% of CSFE^LO^ cells). Moreover, JES6-1 mAb completely blocked the PRBC-stimulated proliferation of CD4^+^ T cells from non-treated mice, while it further increased the response of CD4^+^ T cells from JES6-1-treated mice. These results show that JES6-1 treatment enhances the CD4^+^ T cell response during the chronic phase of *P. chabaudi* infection.

### Effects of JES6-1 treatment on the development of protective immunity to *P. chabaudi* malaria

**Figure 6:**
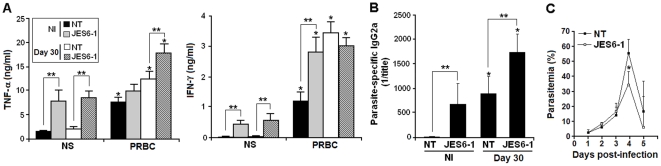
Effects of JES6-1 treatment on the regulation of acquired immune responses to *P. chabaudi* malaria. (A) C57BL/6 mice were treated with JES6-1 mAb on days 0, 2 and 4 p.i. with 10^6^ PRBC. On day 30 p.i., TNF-α and IFN-γ production was evaluated in spleen cell supernatants (mean ± SD, n = 4). (B) Serum titers of parasite-specific IgG2a were determined in the same groups of mice. (C) Parasitemia curves were evaluated in C57BL/6 mice injected with spleen cells from JES6-1-treated mice and non-treated (NT) mice on day 30 p.i. and challenged with 10^8^ PRBC (mean ± SD, n = 4). In A–B, significant differences compared experimental conditions *p<0.05 with non-infected (NI) mice; and **p<0.05 with non-treated (NT) mice. In C, significant differences compared to experimental conditions *p<0.05 with mice transferred with cells from non-treated (NT) mice. Data are representative of two separate experiments.

Because CD4^+^ T cells developed in JES6-1-treated mice have an improved capacity to proliferate when stimulated with PRBC, we sought to verify the effects of *in vivo* JES6-1 treatment on the production of proinflammatory cytokines and parasite-specific IgG2a. In general, JES6-1 treatment increased the non-stimulated (basal) and PRBC-stimulated TNF-α and IFN-γ production in both non-infected and 30-day infected mice (**Fig. 6A**). However, there was no effect on PRBC-stimulated TNF-α secretion by spleen cells from non-infected mice and PRBC-stimulated IFN-γ secretion by spleen cells from 30-day infected mice. On day 30 p.i., the serum titers of parasite-specific IgG2a were significantly higher in JES6-1-treated mice compared to non-treated mice (**Fig. 6B**). Interestingly, similar levels of PRBC-stimulated TNF-α and IFN-γ production and parasite-specific IgG2a were found in non-treated 30-day infected mice and in JES6-1-treated non-infected mice. Both JES6-1-treated and non-treated infected mice efficiently controlled a secondary challenge with 10^8^ PRBC given on day 30 p.i. (data not shown). Additionally, mice injected with spleen cells from 30-day infected mice showed lower parasitemia peaks following infection when cells were taken from JES6-1-treated mice (**Fig. 6C**). We concluded that JES6-1 treatment enhances the Th1 cell response, leading to increased production of TNF-α and parasite-specific IgG2a antibodies and optimizing the protective immunity to *P. chabaudi* malaria.

## Discussion

We have recently proposed that the splenic CD4^+^ T cell response to blood stages of *P. chabaudi* malaria develops in two consecutive phases where conventional CD4^+^ T cells are the main protagonists [13]. The early phase of the response may establish a bridge between innate and acquired immunity, as it rapidly provides large amounts of proinflammatory cytokines and help B cells to secrete polyclonal Ig. The late phase of the response generates a large pool of effector-memory CD4^+^ T cells that cooperate with B cells in the production of parasite-specific high-affinity antibodies and have the capacity to secrete IFN-γ upon stimulation. In the present study, we examined in detail the effects of JES6-1 treatment on the early and late phases of CD4^+^ T cell response to *P. chabaudi* infection.

Initially, we observed that IL-2 is produced during acute infection by activated CD4^+^ T cells expressing high levels of IL-2R α and β chains, as well as the activation markers GITR, mTGF-β, CTLA-4 and CD45RB. The consumption of IL-2 by activated lymphocytes could explain the low levels of this cytokine detected in culture supernatants from human and mouse with acute malaria [8], [25], an interference minimized in our analysis by using a bi-functional anti-IL-2 mAb that binds to the cell surface and competes with IL-2R for capturing the cytokine molecules soon after their release into the extracellular milieu. Corroborating our data, increased production of IL-2 was observed by intracellular staining in blood CD4^+^ T cells from acute malaria patients [26]. Moreover, intracellular IL-2 production coincides with increase of T_reg_ cell population in the spleen during *P. chabaudi* malaria [27].

The concomitant production of IL-2 and expression of high affinity IL-2R by activated CD4^+^ T cells during acute infection implicates this cytokine as a participant in the immune response to *P. chabaudi* malaria. The lack of effect of *in vivo* or *in vitro* JES6-1 treatment in the early CD4^+^ T cell activation, proliferation and IFN-γ production, and consequently in the ability to control acute parasitemia, can be explained by the high expression of CD25 since IL-2 bound to JES6-1 mAb retains the ability to interact with the high-affinity IL-2R [23]. However, it is also possible that the major role of IL-2 during the early CD4^+^ T cell response to *P. chabaudi* infection is to expand the T_reg_ cell population, while the effector CD4^+^ T cell response is independent of IL-2. These possibility is supported by data showing that IL-2 is not required for the initial cycling of T cells, as suggested by previous studies showing that T cell proliferation is not abrogated by anti-IL-2 or anti-IL-2R mAb after strong stimulation through the T cell receptor (TCR) [28], [29]. Moreover, several studies using IL-2- or IL-2R-deficient mice clearly indicate that T cell activation and proliferation occur *in vitro* and *in vivo* independently of all γc-dependent cytokines but are dependent on TCR and costimulatory signals [30], [31], [32], [33].

In agreement with this notion, it has been previously shown that T cell immunity to *P. chabaudi* [34], as well as to other infectious pathogens [35], can be generated in the absence of IL-2. Thus, it is reasonable that intense costimulation makes IL-2 dispensable for T cell activation, a suitable possibility if we consider that IL-2R and the CD28, ICOS and OX40 costimulatory receptors relay intracellular signals through phosphoinositide 3-kinases (PI3K), which culminate in a broad variety of cell biological effects, including proliferation and cytokine synthesis [36]. Accordingly, unpublished data from our laboratory revealed the high expression of ICOS and OX40 during the early CD4^+^ T cell response to *P. chabaudi* malaria (Castillo-Méndez et al., manuscript in preparation). The abundance of costimulatory signals could also cooperate to inactivate T_reg_ cells [37] and consequently enlarge the spectrum of CD4^+^ T cell specificities involved in the early response to infection.

The idea that T_reg_ cell inactivation contributes to the early CD4^+^ T cell response to infection is indirectly supported by our data showing a massive response to parasites in non-infected mice a month after JES6-1 treatment, a condition where T_reg_ cells may have been inactivated by IL-2 deprivation. This increased CD4^+^ T cell response to parasites in JES6-1-treated non-infected mice occurs in terms of both PRBC-stimulated proliferation and IFN-γ production. The mechanism triggering robust CD4^+^ T cell response to PRBC a month after JES6-1 treatment of non-infected mice is currently been investigated by our research group. Our working hypothesis is that depletion of T_reg_ cell population due to IL-2 inhibition results in a long-lasting deficiency in the ability to regulate the immune system, leading to CD4^+^ T cell hyperresponsiveness to PRBC. Although the number of T_reg_ cells per spleen was reestablished on day 20 p.i., the reduced expression of regulatory molecules, such as CTLA-4 and PD-1, may impair their suppressive activity.

The increase in T_reg_ cell population has been previously reported in *P. chabaudi* and *Plasmodium yoelii* infections [38], [39], but its implication in the control of immune responses to malaria is still a matter of debate [5]. Our data extend these findings by showing that IL-2 is partially required for expanding the CD4^+^CD25^+^FoxP3^+^ cell population during *P. chabaudi* malaria. The fact that some increase in CD4^+^CD25^+^FoxP3^+^ cell numbers per spleen still occurred in acutely infected JES6-1-treated mice could also be related to the abundance of costimulatory signals, which have been implicated in T_reg_ cell development and peripheral homeostasis [40]. It is worth noting that, in our analysis, the impaired increase in CD4^+^CD25^+^FoxP3^+^ cell numbers due to JES6-1 treatment does not significantly affect the early CD4^+^ T cell response to parasites, as the only difference observed after JES6-1 treatment in acutely infected mice is a slight augmentation of IFN-γ production.

The effects of JES6-1 treatment *in vitro* drastically change during the infection in which inhibition of CD4^+^ T cell response to parasites is observed at the chronic phase of the disease, but not at the acute phase as discussed above. The results showing the ability of JES6-1 mAb to block *in vitro* CD4^+^ T cell proliferation at the chronic infection demonstrate the effectiveness of this treatment to completely inhibit IL-2 signaling. This shift could be a consequence of the reduction of PI3K-mediated costimulatory signals leading to IL-2 dependency. This process could involve IL-2 induced expression of regulatory molecules, such as PD-1 and CTLA-4, in CD4^+^ T cells [41], [42]. This would increase the threshold for TCR signaling and, as a consequence, restrain the spectrum of CD4^+^ T cell specificities involved in the response and provide an IL-2 driven competitive advantage to parasite-specific CD4^+^ T cells [43]. IL-2 dependency may also allow the control of self (cross) reactive clones by T_reg_ cells, which are thought to operate by scavenging this cytokine [18], [44].

The expansion of T_reg_ cell population during the early CD4^+^ T cell response to infection may be essential for the development of a cohort of T_reg_ cells shaped to modulate the pool of effector-memory and memory CD4^+^ T cells generated during the late phase of the response, by skewing their specificity repertoire towards the recognition of parasite-specific peptides and avoiding the expansion of self (cross) reactive clones. The T_reg_ cell population was still increased on day 20 p.i. indicating that proliferating T_reg_ cells were not eliminated after acute infection. T_reg_ cell inactivation during the acute infection may explain the enhancement of cellular and humoral responses in JES6-1-treated mice during the chronic infection, which results in the improved capacity of spleen cells to transfer protection to naïve mice. Spleen cells from JES6-1-treated mice conferred a substantial protection (∼40% reduction in parasitemia) if we consider the high parasite inoculum used in mouse challenge, and we can envisage that concomitant treatment with additional immunotherapies such as anti-IL-10 mAb could further improve parasite control.

Another possible explanation for the increase of immune response in JES6-1-treated chronic mice is related to the fact that, in some particular conditions, IL-2/JES6-1 mAb complexes can potentiate IL-2 signaling and amplify the effector T cell function [24]. Although this phenomenon is observed following administration of premixed 2∶1 molar ratio of IL-2/JES6-1 mAb complexes, the possibility that similar conditions occur *in vivo* cannot be discarded. It should be noted, however, that in our experiments JES6-1 treatment results in substantial CD4^+^ T cell activation in non-infected mice, whereas IL-2/JES6-1 treatment leads to expansion of the T_reg_ cell population and, in consequence, to reduction of CD4^+^ and CD8^+^ T cell responses to *Plasmodium berghei* infection [24] and to increase in parasitemia during *P. chabaudi* infection [27]. These opposite effects indicate that JES6-1 mAb is responsible for very different outcomes when administered alone or coupled with two molecules of IL-2, an artificial condition that may cause the approximation of two IL-2Rα chains and result in cell signaling independent of the β chain.

This work reinforces the idea that the CD4^+^ T cell response to *P. chabaudi* malaria develops in two distinct phases wherein JES6-1 treatment impairs the expansion of T_reg_ cell population at the early phase and enhances the Th1 cell response at the late phase. This study may help to understand the molecular mechanisms involved in the immune response to *Plasmodium* and contribute to efforts aiming to manipulate this response to ameliorate the outcome of the disease.

## Materials and Methods

### Mice, parasites and infection

Six to eight-week-old C57BL/6 female mice were bred under pathogen-free conditions at the Isogenic Mice Facility, ICB-USP, São Paulo, Brazil. *Plasmodium chabaudi* AS was maintained as previously described [9]. Mice were infected intraperitoneally and parasitemias were determined by microscopic examination of Giemsa stained blood smears.

### Ethics Statements

All procedures were in accordance with national regulations of ethical guidelines for mice experimentation and welfare of the conselho nacional de saúde and colégio brasileiro em experimentação animal (cobea), brazil, the protocols being approved by the health animal comitte (comissão de ética no uso de animais - ceua - icb/usp) of the instituto de ciências biomédicas of the universidade de são paulo, são paulo, brazil, qith permit number 0019/2005 and 0036/2007.

### Anti-IL-2 mAb treatment

Mice were treated intraperitoneally with rat anti-IL-2 (JES6-1A12) mAb (1 mg/mouse/day) on days 0, 2 and 4 of infection. Spleen cells were cultured *in vitro* with 0.2 µg/ml anti-IL-2 mAb.

### Spleen cell suspensions

Spleen cells were cultured in RPMI 1640 supplemented with penicillin (100 U/ml), streptomycin (100 µg/ml), 2-mercaptoethanol (50 µM), L-glutamine (2 mM), sodium pyruvate (1 mM) and 3% heat-inactivated fetal calf serum (FCS). All supplements were purchased from Life Technologies. Cell numbers were determined with a Neubauer chamber.

### Spleen cell phenotyping

Spleen cells (1×10^6^) were stained with FITC-, PE-, PerCP- or APC-labeled mAb to CD4 (H129.19), CD25 (PC61), CD45RB (16A), CD62L (MEL-14), CD122 (TMβ1), CD69 (H1-2F3), CD44 (IM7) and CTLA-4 (UC-4F10-11) from BD Pharmingen. Biotinylated mAb to mTGF-β (G766B) from Promega was detected with FITC-labeled streptavidin (BD Pharmingen). Unlabeled mAb to GITR (108619) from RD System was detected with FITC-labeled polyclonal anti-rat Ig from BD Pharmingen. Cells were analyzed by flow cytometry using a FACScalibur with CELLQUEST software (Becton Dickinson). The percentages of large cells were determined by analyzing forward light scatter (FSC), using gates defined in histograms of non-activated splenocytes.

### Intracellular staining of Foxp3

Spleen cells (1×10^6^) were stained with anti-CD4 mAb and fixed with Cytofix/Cytoperm (BD Bioscience) for 30 min at room temperature. Cells were permeabilized with 1% formaldehyde for 30 min, centrifuged and washed twice in PBS containing 5% FCS and 0.1% NaN_3_. After incubation for 2 h with FITC conjugated anti-mouse/rat Foxp3 mAb, cells were washed and analyzed by flow cytometry.

### IL-2 secretion assay

The IL-2 PE cytokine secretion assay was performed according to the manufacturer's recommendations (BD Biosciences). This assay uses a bi-functional mAb capable of binding CD45 on one arm and IL-2 on the other arm. Spleen cells (2×10^6^) were incubated with the bi-functional mAb for 45 min at 37°C in 5% CO_2_. The presence of IL-2 bound to bi-functional mAb was detected with PE-labeled anti-IL-2 mAb. As a positive control, spleen cells (2×10^6^) from non-infected mice were cultured *in vitro* for 3 h with anti-CD3 mAb (BD Pharmingen). The analyses were performed by flow cytometry.

### Proliferation assay

The proliferative CD4^+^ cell response was measured as previously described [45]. Briefly, 6×10^6^ cells/ml in PBS with 0.1% BSA were incubated with 5,6-carboxy fluorescein succinimidyl ester (CFSE; Molecular Probes) at a final concentration of 5 µM for 20 min at 37°C. Cells (1×10^6^) were cultured in 96-well plates (Costar) with PRBC (3×10^6^) or medium alone for 72 h at 37°C in 5% CO_2_. After incubation, cells were stained with PE-labeled mAb to CD4 and analyzed by flow cytometry.

### Multicytokine assessment

IFN-γ and TNF-α were quantified simultaneously by a cytometric bead array (CBA; BD Pharmingen) of supernatants obtained from the same cultures used in the proliferation assay. This technique uses flow cytometry to measure soluble analytes in a particle-based immunoassay and was carried out according to the manufacturer's instructions (BD Pharmingen). The lower limit of detection for all cytokines in this assay was 20 pg/ml.

### Intracellular staining for IFN-γ and IL-17

For intracellular IFN-γ and IL-17, spleen cells (1×10^6^) were cultured with GolgiStop overnight at 37°C in 5% CO_2_ according to the manufacturer's instructions (BD Pharmingen). After washing, cells were surface stained with FITC- or Cy-Chrome-labeled mAb to CD4 and CD8. Cells were then fixed with Cytofix/Cytoperm buffer and incubated with PE-labeled mAb to IFN-γ and IL-17 diluted in Perm/Wash buffer. The analysis was performed by flow cytometry.

### Parasite-specific ELISA

Anti-*P. chabaudi* antibodies were quantified by ELISA as previously described [46]. In brief, 96-well flat-bottom microtiter plates (Costar) were coated overnight (4°C) with a total parasite extract (8 µg/ml). Plates were saturated with 1% BSA (bovine serum albumin) for 1 h. After washing, wells were incubated with 100 µl mouse serum (diluted from 1∶50 to 1∶6400) for 90 min at room temperature. Assays were developed with goat anti-mouse IgM or IgG2a peroxidase conjugated antibodies (Southern Biotechnology Associates) for 1 h, followed by 100 µl/well of TMB (tetra-methyl-benzidine) (Zymed) for 15 min, and the absorbance was quantified with a Spectra Max 190 spectrophotometer (Molecular Devices) at 650 nm. The antibody level in each serum sample was expressed as the reciprocal of the endpoint titer, which was defined in serial dilutions as the lowest dilution with a background optical density (O. D.).

### Statistical analysis

Statistical analysis was generally performed with t-test and Mann Whitney test using Graph Pad Prism 4 software. Differences between two groups were considered significant when the *p* value was <0.05 (5%).
